# Characteristics of Pediatric Mild Traumatic Brain Injury and Recovery in a Concussion Clinic Population

**DOI:** 10.1001/jamanetworkopen.2020.21463

**Published:** 2020-11-16

**Authors:** Philip E. Rosenbaum, Christopher Locandro, Sara P. D. Chrisman, Meeryo C. Choe, Rachel Richards, Christina Pacchia, Lawrence J. Cook, Frederick P. Rivara, Gerard A. Gioia, Christopher C. Giza

**Affiliations:** 1 David Geffen School of Medicine, Department of Neurosurgery, University of California, Los Angeles; 2 Steve Tisch BrainSPORT Program, University of California, Los Angeles; 3 Department of Pediatrics University of Utah, Salt Lake City; 4 Center for Child Health, Behavior and Development, Seattle Children’s Research Institute, Seattle, Washington; 5 Department of Pediatrics, University of Washington, Seattle; 6 Harborview Injury Prevention and Research Center, Seattle, Washington; 7 David Geffen School of Medicine, Department of Pediatrics, UCLA Mattel Children’s Hospital, Los Angeles, California; 8 Departments of Pediatrics and Psychiatry and Behavioral Sciences, George Washington University School of Medicine, Washington, District of Columbia; 9 Children’s National Hospital, Rockville, Maryland

## Abstract

**Importance:**

Pediatric mild traumatic brain injury (TBI) and concussion are a public health challenge with up to 30% of patients experiencing prolonged recovery. Pediatric patients presenting to concussion clinics often have ongoing impairments and may be at increased risk for persistent symptoms. Understanding this population is critical for improved prognostic estimates and optimal treatment.

**Objective:**

To describe pediatric patients presenting to concussion clinics and characterize factors associated with their recovery.

**Design, Setting, and Participants:**

This prospective cohort study included patients enrolled at multicenter concussion specialty clinics from the Four Corners Youth Consortium from December 2017 to July 2019, with up to 12-month follow-up. Patients were eligible if they were aged 5 to 18.99 years with a diagnosis of mild TBI or concussion presenting to participating clinics within 8 weeks of injury. Patients were excluded if the patient or their parents were unable to read or sign the consent document, or if the patient had a Glasgow Coma Scale score less than 13 or a penetrating injury. Data were analyzed from February 2019 to April 2020.

**Exposures:**

Diagnosis of mild TBI or concussion.

**Main Outcomes and Measures:**

This study used National Institute of Neurological Disorders and Stroke common data elements, including data on demographic characteristics, injury details, history, neurological and neuropsychological assessments, and treatment.

**Results:**

A total of 600 patients were consecutively enrolled, among whom 324 (54.0%) were female and 435 (72.5%) were adolescents (ie, aged 13-18 years). A higher proportion of girls and women (248 patients [76.5%]) were adolescents compared with boys and men (187 patients [67.8%]) (*P* = .02), and girls and women reported significantly more preexisting anxiety compared with boys and men (80 patients [26.7%] vs 46 patients [18.7%]; *P* = .03). Significantly more adolescents reported preexisting migraines compared with preadolescents (82 patients [20.9%] vs 15 patients [10.9%]; *P* = .01). Girls and women recovered more slowly than boys and men (persistent symptoms after injury: week 4, 217 patients [81.6%] vs 156 patients [71.2%]; week 8, 146 patients [58.9%] vs 89 patients [44.3%]; week 12, 103 patients [42.6%] vs 58 patients [30.2%]; *P* = .01). Patients with history of migraine or anxiety or depression recovered more slowly than those without, regardless of sex.

**Conclusions and Relevance:**

These findings suggest that identification of subgroups of pediatric patients with mild TBI or concussion at risk for prolonged recovery could aid in better prognostic estimates and more targeted treatment interventions.

## Key Points

**Question:**

What factors are associated with recovery in pediatric patients with mild traumatic brain injury or concussion?

**Findings:**

In this cohort study of 600 pediatric patients presenting to concussion clinics in 3 centers, girls and women experienced slower recovery and were more likely to have preexisting anxiety compared with boys and men. Independent of sex, patients with anxiety or depression or migraine recovered more slowly than those without these comorbidities.

**Meaning:**

These findings suggest that factors, such as sex and comorbidities, in pediatric patients with mild traumatic brain injury are essential for accurate prognostic estimates and identifying targets for treatment.

## Teaser

This cohort study examines patient characteristics associated with recovery time in pediatric patients with mild traumatic brain injury treated at concussion specialty clinics.

## Introduction

It is estimated that more than 830 000 pediatric patients with traumatic brain injury (TBI) present to emergency departments (EDs) each year in the US.^1^ Mild TBI, including concussion, accounts for at least 75% of all TBIs reported in the US.^2^ A 2015 study^3^ of pediatric concussion in an ED cohort found that adolescents aged 12 to 17 years had a higher incidence of concussion compared with younger children, but relatively few studies address children in the 5 to 12 years age range. Pediatric patients with mild TBI present to a variety of medical settings, with a 2016 study^4^ reporting 82% of patients first seen in primary care, 11% of patients presenting to the ED, and 5.2% of patients presenting to a specialty clinic. Therefore, mild TBI incidence based solely on ED visits underestimates the number of individuals with mild TBI. A more complete understanding of the full range of needs of youth with mild TBI requires the study of all mechanisms across all age ranges and clinical settings, including the outpatient clinic population.

Although most children with mild TBI recover relatively rapidly, 10% to 30% have persistent postconcussion symptoms (PPCS) lasting longer than 4 to 12 weeks,^5,6,7^ and such prolonged recovery can interfere with academics and quality of life.^8^ A large population-based study of pediatric TBI in Sweden^9^ concluded that mild TBI in youth was associated with adverse outcomes in adulthood, and recurrent TBI and age-at-injury were important factors associated with outcome. These pediatric patients with mild TBI with prolonged recovery are of major concern, as they experience greater disability and require more medical resources. Therefore, better defining the problem of mild TBI, PPCS, and longer-term outcomes in children and adolescents is an important public health challenge and well suited to large, prospective cohorts recruited from pediatric mild TBI clinics.

Factors that may increase risk for PPCS and prolonged recovery include age, sex, and premorbid conditions. However, much of the research regarding mild TBI recovery comes from emergency or acute care cohorts using relatively short outcome windows (ie, ≤1 month).^7,10,11^ Currently, there is no widely accepted definition or time interval for PPCS in children,^12^ further challenging clinicians’ ability to identify and treat these patients. Moreover, factors associated with PPCS and prolonged recovery may differ in different clinical populations (eg, athletes vs nonathletes, adolescents vs children), clinical settings (eg, specialty concussion clinics having a higher proportion of subacute or chronic patients with PPCS than primary care clinics) and time intervals (eg, 1 month vs ≥3 months after injury).

The Four Corners Youth Consortium (4CYC) was formed through collaboration among academic institutions with expertise and multidisciplinary programs focused on pediatric mild TBI clinical care and research.^13^ The 4CYC is unique in that we are focused on the population of youth seen in subspecialty mild TBI and concussion clinics and have been able to capture longer follow-up after injury. This study examined trajectories of symptom recovery in patients presenting to pediatric mild TBI clinics. We hypothesized age, sex, and premorbid factors are associated with mild TBI recovery and persistence of symptoms in this specialty clinic population.

## Methods

This study was reviewed and approved by a single institutional review board at the University of Utah. Informed consent was obtained from the parent, guardian, or patient if the patient was aged 18 years and assent was obtained from children aged 5 to 17.99 years in accordance with site-specified institutional review board compliance regulations. Consent was preferentially obtained in person but could optionally be obtained electronically through email and telephone communication. For patients who did not consent to be contacted for follow-up, demographic and initial clinical data were extracted from the electronic health record under an institutional review board–approved waiver of consent. Waiver of consent was approved to maximize completeness of the data set and permit extraction of deidentified medical information, given the low-risk nature of this registry. This study follows the Strengthening the Reporting of Observational Studies in Epidemiology (STROBE) reporting guideline.

### The 4CYC Concussion Registry

The 4CYC is a multicenter collaborative that aims to build a comprehensive evidence base to promote behaviors that improve brain health among youth.^13^ Three contributing member sites, Children’s National Hospital, Seattle Children’s Hospital–University of Washington, and UCLA Mattel Children’s Hospital, have built a prospective, observational registry of pediatric mild TBI. The University of Utah Data Coordinating Center joined the 4CYC investigators and constructed a database using National Institute of Neurological Disorders and Stroke common data elements.

### Enrollment and Consent

From December 2017 to July 2019, the registry enrolled pediatric patients aged 5 to 18.99 years presenting with mild TBI within 8 weeks of injury. Patients who enrolled at age 18 years were followed through recovery even if recovery transcended their 18th year. Patients were excluded if the patient or their parent was unable to read or sign the consent document, or if the patient had an initial Glasgow Coma Scale score less than 13 or a penetrating injury.

### Clinical Measures

The study used National Institute of Neurological Disorders and Stroke common data elements from the guidelines for pediatric TBI, mild TBI, and sports concussion.^14,15^ Sites collected demographic characteristics, injury details, medical history, and clinical neurological assessments directly from the patient or parent and any available prior medical records as part of regular clinic care. The patient- or parent-reported past medical history was self-reported during an interview conducted by a licensed health care practitioner and included the patient’s preexisting comorbidities, including attention-deficit/hyperactivity disorder, anxiety, depression, learning disabilities, migraines, sleep problems, and seizures or epilepsy. This information was gathered as part of standard clinical care. Data were extracted from the electronic health record into a REDCap database (Vanderbilt University).

### Follow-up and Patient Recovery

Contact information was collected from patients who consented to receive follow-up surveys. Surveys were administered directly from REDCap by text, email, or telephone call, as preferred by the patient’s parent. The follow-up was performed every 3 months following the date of injury until the parent indicated the patient had fully recovered from the injury. Recovery was defined as “all of the symptoms that were caused BY THE INJURY have GONE AWAY and DO NOT RETURN when doing activities (physical or mental), such as exercise or studying for school.” Some patients followed-up in clinic as part of the standard clinical care; for these patients, the date of recovery was determined by both clinical examination and interview at the time of follow-up. For patients with both parent- and clinician-reported recovery, the clinician-reported date was used for analysis.

### Statistical Analysis

Age groups were designated as preadolescent (ie, age 5-12.99 years) and adolescent (ie, age 13-18.99 years). Patient and injury characteristics were summarized by age group and sex using frequencies and percentages for categorical variables or median and interquartile range for continuous variables. Differences in patient and injury characteristics between the younger and older age groups were tested using Fisher exact test, with the exception of the number of comorbidities, for which the Kruskal-Wallis test was used. Kaplan-Meier curves were used to compare time to recovery by age groups, sex, number of comorbidities, prior TBI, migraine history, and history of emotional distress (defined as anxiety and/or depression). Patients without a documented date of recovery were considered censored at the date of the last known clinic visit or follow-up survey. Log-rank tests were used to compare recovery curves.

All hypothesis tests were conducted against a 2-sided alternative. *P* values were considered statistically significant when less than .05. Analyses were performed using SAS statistical software version 9.4 (SAS Institute). Data were analyzed from February 2019 to April 2020.

## Results

### Demographic Characteristics

A total of 600 patients were enrolled in the study, among whom 324 (54.0%) were female and 435 (72.5%) were adolescents. Overall demographic characteristics of the 4CYC cohort and breakdown by sex are summarized in Table 1. Compared with boys and men, a greater proportion of girls and women were adolescents (187 patients [67.8%] vs 248 patients [76.5%]; *P* = .02). Most patients were non-Hispanic (475 patients [87.8%]) and White (375 patients [75.6%]). Medicaid or state Child Health Insurance Plan covered only 91 patients (15.3%). Medicaid covered more of the boys and men in our cohort compared with girls and women. Preinjury, most patients were in regular school. Parental education was high, with most parents having a college degree or above, and almost half having a masters or doctoral degree (Table 1). A total of 372 parents (62.0%) consented to follow-up. Analysis between those with follow-up and those who did not consent for follow-up showed no significant differences (eTable 1 in the Supplement). Of patients who consented for follow-up, 293 (78.8%) actually responded to follow-up surveys. Patients who responded to follow-up were more likely to have comorbidities than those who did not; however, they did not differ otherwise (eTable 2 in the Supplement).

**Table 1.** Patient Characteristics by Sex

Characteristic	Patients, No./Total No. (%)			*P* value
	Male (n = 276)	Female (n = 324)	Overall (N = 600)	
Age at injury, y				
5-12	89/276 (32.2)	76/324 (23.5)	165/600 (27.5)	.02^a^
13-18	187/276 (67.8)	248/324 (76.5)	435/600 (72.5)	
Ethnicity				
Not Hispanic or Latino	222/250 (88.8)	253/291 (86.9)	475/541 (87.8)	.60^a^
Hispanic or Latino	28/250 (11.2)	38/291 (13.1)	66/541 (12.2)	
Race				
American Indian or Native Hawaiian	4/229 (1.7)	3/267 (1.1)	7/496 (1.4)	.83^a^
Asian	12/229 (5.2)	14/267 (5.2)	26/496 (5.2)	
African American	23/229 (10.0)	21/267 (7.9)	44/496 (8.9)	
White	172/229 (75.1)	203/267 (76.0)	375/496 (75.6)	
Multiracial	18/229 (7.9)	26/267 (9.7)	44/496 (8.9)	
Insurance type				
Medicaid or state Child Health Insurance Plan	52/276 (18.8)	39/320 (12.2)	91/596 (15.3)	.045^a^
Commercial	217/276 (78.6)	275/320 (85.9)	492/596 (82.6)	
Medicare	2/276 (0.7)	0/320 (0.0)	2/596 (0.3)	
None or self-pay	5/276 (1.8)	6/320 (1.9)	11/596 (1.8)	
Highest parent education level				
No education	0/160 (0)	0/209 (0)	0/369 (0)	.79^a^
<High school graduate	6/160 (3.8)	5/209 (2.4)	11/369 (3.0)	
High school graduate or GED	6/160 (3.8)	10/209 (4.8)	16/369 (4.3)	
Vocational school or some college	15/160 (9.4)	24/209 (11.5)	39/369 (10.6)	
College degree	54/160 (33.8)	76/209 (36.4)	130/369 (35.2)	
Master’s or doctoral degree	79/160 (49.4)	94/209 (45.0)	173/369 (46.9)	
Educational services received prior to concussion				
Special education (IEP, 504 Plan)	53/255 (20.8)	47/302 (15.6)	100/557 (18.0)	.12^a^
Regular education	202/255 (79.2)	255/302 (84.4)	457/557 (82.0)	
Patient medical history				
ADHD	42/239 (17.6)	40/296 (13.5)	82/535 (15.3)	.23^a^
Anxiety	46/246 (18.7)	80/300 (26.7)	126/546 (23.1)	.03^a^
Depression	19/240 (7.9)	39/293 (13.3)	58/533 (10.9)	.05^a^
Learning disability	26/235 (11.1)	23/294 (7.8)	49/529 (9.3)	.23^a^
Sleep problems	20/238 (8.4)	38/291 (13.1)	58/529 (11.0)	.10^a^
Seizures	3/237 (1.3)	8/288 (2.8)	11/525 (2.1)	.36^a^
Migraines	35/239 (14.6)	62/291 (21.3)	97/530 (18.3)	.06^a^
Prior concussion	135/270 (50.0)	157/319 (49.2)	292/589 (49.6)	.87^a^
Comorbidities, No. (excluding seizures)				
0	66/207 (31.9)	69/264 (26.1)	135/471 (28.7)	.06^b^
1	88/207 (42.5)	110/264 (41.7)	198/471 (42.0)	
2	30/207 (14.5)	40/264 (15.2)	70/471 (14.9)	
≥3	23/207 (11.1)	45/264 (17.0)	68/471 (14.4)	
Family medical history				
Migraines	84/218 (38.5)	117/270 (43.3)	201/488 (41.2)	.31^a^
Depression	73/212 (34.4)	81/258 (31.4)	154/470 (32.8)	.49^a^
Anxiety	82/210 (39.0)	89/259 (34.4)	171/469 (36.5)	.34^a^
ADHD	53/203 (26.1)	55/254 (21.7)	108/457 (23.6)	.27^a^
Learning disability	37/204 (18.1)	44/254 (17.3)	81/458 (17.7)	.90^a^
Family comorbidities, No.				
0	65/190 (34.2)	96/238 (40.3)	161/428 (37.6)	.64^b^
1	55/190 (28.9)	55/238 (23.1)	110/428 (25.7)	
2	33/190 (17.4)	31/238 (13.0)	64/428 (15.0)	
≥3	37/190 (19.5)	56/238 (23.5)	93/428 (21.7)	

Abbreviations: ADHD, Attention Deficit Hyperactivity Disorder; GED, General Educational Development; IEP, individualized education program.

^a^ Fisher exact test.

^b^ Kruskal-Wallis test.

Patient preinjury medical history is reported by sex in Table 1 and by age group in Table 2. Significantly more girls and women reported preexisting anxiety than did boys and men (80 patients [26.7%] vs 46 patients [18.7%]; *P* = .03). No differences were found between sexes in the preinjury diagnoses of attention-deficit/hyperactivity disorder, migraine, depression, or learning disabilities as well as a count of total comorbidities. Adolescents, compared with preadolescents, were more likely to report a diagnosis of migraines (82 patients [20.9%] vs 15 patients [10.9%]; *P* = .01) and a history of prior concussion (234 patients [54.8%] vs 15 patients [10.9%]; *P* < .001). Adolescents had more total comorbidities than preadolescents (eg, ≥3 comorbidities: 53 patients [15.1%] vs 15 patients [12.5%]; *P* = .008).

**Table 2.** Patient Characteristics by Age

Characteristic	Patients, No./Total No. (%)			*P* value
	Age, y		Overall (N = 600)	
	5-12 (n = 165)	13-18 (n = 435)		
Sex				
Male	89/165 (53.9)	187/435 (43.0)	276/600 (46.0)	.02^a^
Female	76/165 (46.1)	248/435 (57.0)	324/600 (54.0)	
Ethnicity				
Not Hispanic or Latino	128/146 (87.7)	347/395 (87.8)	475/541 (87.8)	>.99^a^
Hispanic or Latino	18/146 (12.3)	48/395 (12.2)	66/541 (12.2)	
Race				
American Indian or Native Hawaiian	1/131 (0.8)	6/365 (1.6)	7/496 (1.4)	.67^a^
Asian	9/131 (6.9)	17/365 (4.7)	26/496 (5.2)	
African American	14/131 (10.7)	30/365 (8.2)	44/496 (8.9)	
White	97/131 (74.0)	278/365 (76.2)	375/496 (75.6)	
Multiracial	10/131 (7.6)	34/365 (9.3)	44/496 (8.9)	
Insurance type				
Medicaid or state Child Health Insurance Plan	28/163 (17.2)	63/433 (14.5)	91/596 (15.3)	.44^a^
Commercial	134/163 (82.2)	358/433 (82.7)	492/596 (82.6)	
Medicare	0/163 (0.0)	2/433 (0.5)	2/596 (0.3)	
None or self-pay	1/163 (0.6)	10/433 (2.3)	11/596 (1.8)	
Highest parent education level				
No education	0/106 (0)	0/263 (0)	0/369 (0)	.42^a^
<High school graduate	2/106 (1.9)	9/263 (3.4)	11/369 (3.0)	
High school graduate or GED	4/106 (3.8)	12/263 (4.6)	16/369 (4.3)	
Vocational school or some college	7/106 (6.6)	32/263 (12.2)	39/369 (10.6)	
College degree	43/106 (40.6)	87/263 (33.1)	130/369 (35.2)	
Master’s or doctoral degree	50/106 (47.2)	123/263 (46.8)	173/369 (46.9)	
Educational services received prior to concussion				
Special education (IEP, 504 Plan)	31/146 (21.2)	69/411 (16.8)	100/557 (18.0)	.26^a^
Regular education	115/146 (78.8)	342/411 (83.2)	457/557 (82.0)	
Patient medical history				
ADHD	26/141 (18.4)	56/394 (14.2)	82/535 (15.3)	.28^a^
Anxiety	32/146 (21.9)	94/400 (23.5)	126/546 (23.1)	.73^a^
Depression	13/143 (9.1)	45/390 (11.5)	58/533 (10.9)	.53^a^
Learning disabilities	12/142 (8.5)	37/387 (9.6)	49/529 (9.3)	.87^a^
Sleep problems	21/143 (14.7)	37/386 (9.6)	58/529 (11.0)	.12^a^
Seizures	3/140 (2.1)	8/385 (2.1)	11/525 (2.1)	>.99^a^
Migraines	15/138 (10.9)	82/392 (20.9)	97/530 (18.3)	.01^a^
Prior concussions	58/162 (35.8)	234/427 (54.8)	292/589 (49.6)	<.001^a^
Comorbidities, No. (excluding seizures)				
0	48/120 (40.0)	87/351 (24.8)	135/471 (28.7)	.008^b^
1	43/120 (35.8)	155/351 (44.2)	198/471 (42.0)	
2	14/120 (11.7)	56/351 (16.0)	70/471 (14.9)	
≥3	15/120 (12.5)	53/351 (15.1)	68/471 (14.4)	
Family medical history				
Migraines	48/132 (36.4)	153/356 (43.0)	201/488 (41.2)	.21^a^
Depression	41/127 (32.3)	113/343 (32.9)	154/470 (32.8)	.91^a^
Anxiety	41/127 (32.3)	130/342 (38.0)	171/469 (36.5)	.28^a^
ADHD	34/128 (26.6)	74/329 (22.5)	108/457 (23.6)	.39^a^
Learning disability	23/124 (18.5)	58/334 (17.4)	81/458 (17.7)	.78^a^
Family comorbidities, No.				
0	46/116 (39.7)	115/312 (36.9)	161/428 (37.6)	.27^b^
1	33/116 (28.4)	77/312 (24.7)	110/428 (25.7)	
2	17/116 (14.7)	47/312 (15.1)	64/428 (15.0)	
≥3	20/116 (17.2)	73/312 (23.4)	93/428 (21.7)	

Abbreviations: ADHD, Attention Deficit Hyperactivity Disorder; GED, General Educational Development; IEP, individualized education program.

^a^ Fisher exact test.

^b^ Kruskal-Wallis test.

### Injury Characteristics

Patients presented to a clinic a median (interquartile range) of 16.0 (8.0-29.0) days after injury. Injury characteristics are presented by sex in eTable 3 in the Supplement and by age group in eTable 4 in the Supplement. Girls and women were more likely to present with stable or worsening symptoms over the course of the first week, while boys and men were more likely than girls and women to have improving symptoms within the first week. There were no other significant sex differences in injury mechanism or characteristics.

Acute injury severity surrogates, including neuroimaging anomalies, amnesia, and loss of consciousness, are reported in eTable 5 in the Supplement. In this cohort, relatively few patients underwent acute or subacute neuroimaging, consistent with existing clinical guidelines for pediatric mild TBI.^16^

Most injuries (452 injuries [75.3%]) came from sports or recreation, followed by being struck by or against an object or person and falling (nonsport). When examining the cause of injury by age, adolescents were more likely to suffer a sports- or recreation-related injury than preadolescents. Preadolescents were more likely to sustain a nonsport-related accident or be struck by an object or person than were adolescents. There were no other age group differences in injury characteristics (eTable 4 in the Supplement).

### Recovery

**Figure 1.**
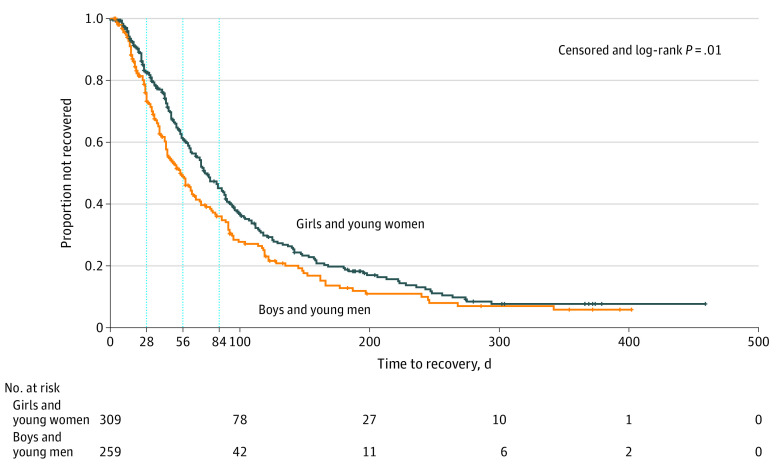
Kaplan-Meier Curves for Time to Recovery by Sex

**Figure 2.**
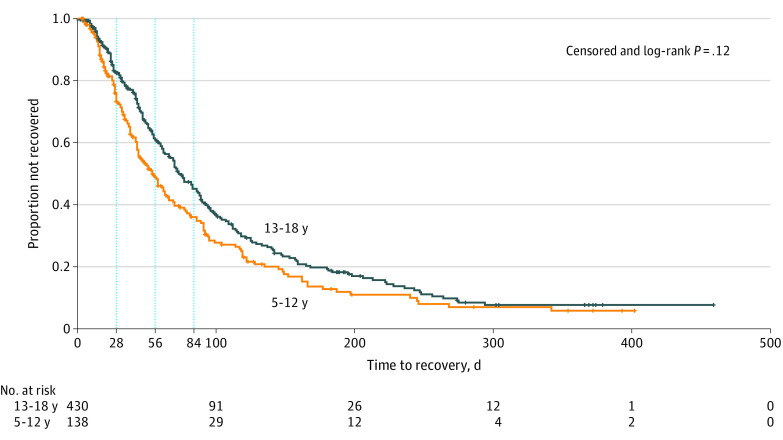
Kaplan-Meier Curves for Time to Recovery by Age Category

**Figure 3.**
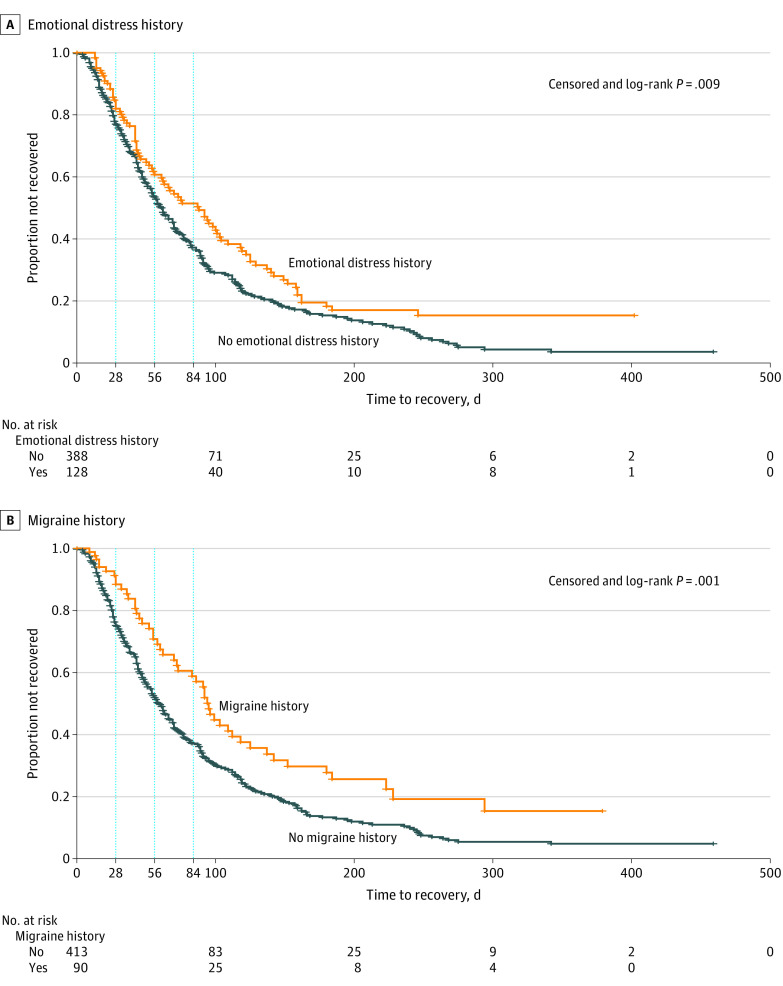
Kaplan-Meier Curves for Time to Recovery by Emotional Distress History or Migraine History

Girls and women recovered at a slower rate than boys and men (persistent symptoms after injury: week 4, 217 patients [81.6%] vs 156 patients [71.2%]; week 8, 146 patients [58.9%] vs 89 patients [44.3%]; week 12, 103 patients [42.6%] vs 58 patients [30.2%]; *P* = .01) (Figure 1). There was no significant difference in persistent symptoms in adolescents vs preadolescents (Figure 2). Patients who reported preinjury history of emotional distress (ie, anxiety or depression) recovered more slowly than those without (persistent symptoms after injury: week 4, 89 patients [80.9%] vs 251 patients [75.6%]; week 8, 59 patients [57.8%] vs 156 patients [50.5%]; week 12, 48 patients [48.0%] vs 99 patients [33.3%]; *P* = .009) (Figure 3A). Patients with a migraine history had more persistent symptoms than those without migraine (persistent symptoms after injury: week 4, 62 patients [87.3%] vs 266 patients [73.9%]; week 8, 42 patients [67.7%] vs 165 patients [49.0%]; week 12, 34 patients [55.7%] vs 108 patients [33.2%]; *P* = .001) (Figure 3B). Neither overall burden of comorbidities nor history of prior concussion showed a significant association with recovery. One post hoc analysis showed prior emotional distress or migraine was associated with slower recovery irrespective of sex (eTable 6 and eTable 7 in the Supplement). Acute injury severity surrogates of neuroimaging with anomalies, amnesia, or loss of consciousness were not associated with prolonged recovery.

## Discussion

This prospective multicenter cohort study describes mild TBI recovery outcomes of a large cohort of patients presenting to subspecialty clinics, examining sex and age associations of mild TBI recovery profiles. This 4CYC study further demonstrates the ability to obtain a comprehensive and clinically useful pediatric mild TBI data set in the course of a usual multidisciplinary clinic visit.

This 4CYC cohort study examines the recovery characteristics of an important group of patients with mild TBI presenting to subspecialty care. These youths represent a subgroup at greater risk of experiencing prolonged recovery and PPCS than youths presenting in more acute settings, with more than 70% of youths in this study having symptoms lasting longer than 1 month and 40% of youths still symptomatic at 3 months. A better understanding of this group’s characteristics is a major public health priority for providing improved prognostic estimates, more accurate assessment, and timely intervention.

Studying children and adolescents from outpatient subspecialty concussion clinics captures a different sample of patients than those from the ED^4,17^ or athletics.^18,19^ A 2013 multisite study of mild TBI based in the ED^20^ recruited patients with more severe or highly symptomatic initial injuries, prompting early presentation. Conversely, a 2010 study of youth sport-related mild TBI^18^ acquired data through school-based athletic trainers, resulting in patients with injuries who often do not present for care at an outpatient concussion clinic or ED. Studies in ED patients and youths with sports-related mild TBI have reported a relatively rapid recovery in most individuals, with a much smaller proportion of patients reporting ongoing symptoms at 1 month^7,21^ or 3 months.^5,6,20,22,23,24^

Another important characteristic of the 4CYC cohort was that a substantial proportion of patients were preadolescent, while many earlier studies, particularly in sports-related TBI, have focused primarily on high school–aged youth.^18,25,26,27^ The inclusion of younger children with mild TBI can help us determine age-specific differences in symptom presentation and recovery. While demographic studies of pediatric TBI have shown a 2-to-1 predominance of boys,^25^ our 4CYC cohort was comprised of almost equal numbers of boys and girls, with an increasing proportion of girls and women (>57%) in the adolescent age range, a factor that this study suggests is essential for prognostic estimates.

Our sample of patients treated in specialty mild TBI clinics had higher socioeconomic status than the general population, with low rates of Medicaid insurance and high rates of parental education, similar to what has been reported in a study by Copley et al.^28^ A more socioeconomically balanced sample is necessary in future work to ensure that the recovery characteristics of youth with lesser financial and educational resources are also well defined.

### Association of Sex and Age

An age by sex difference was evident in our sample, with a larger proportion of girls and women in the adolescent group. Adolescent girls and women have been shown in ED and sports studies to be at higher risk for PPCS.^7,29^ Prolonged recovery was also seen in girls and women in this 4CYC cohort. Many factors have been ascribed to the sex associations of concussion risk and recovery, including neck strength, hormonal differences, comorbidities with a sex predominance, symptom reporting, and social biases. While girls and women took longer to recover in this concussion clinic cohort, differences could not entirely be ascribed to presence of selected comorbidities, suggesting other underlying biological or social determinants.

It is known that onset of migraine,^30^ anxiety,^31^ and depression^32^ is typically in adolescence. However, in the 4CYC population, only migraine and history of prior concussion showed significant age differences. Prior studies have reported mixed results in determining whether the immature brain is more susceptible or more resilient to TBI and concussion, with a 2018 study^33^ showing younger children to be more susceptible and other studies finding adolescence as a period of greater risk for developing prolonged problems.^7,9^ While there was significant association of sex with recovery time, there was no significant difference in recovery time in adolescents compared with preadolescents.

### Comorbidities and Recovery

The interaction between sex and select comorbidities has often been implicated in mild TBI recovery, including mental health problems, such as preexisting attention-deficit/hyperactivity disorder, learning disability, anxiety, depression, sleep problems, or migraines, or prior history of concussion.^5,7,20,34,35^ Although the 4CYC study did not find an association with all of these factors, girls and women were more likely than boys and men to have a history of anxiety. The Centers for Disease Control and Prevention reports rates of clinician-diagnosed anxiety and depression in children aged 3 to 17 years without mild TBI at 7% for anxiety and 3% for depression.^36^ However, other epidemiological studies show much higher rates of anxiety (30%) and mood disorders (11%) in adolescents aged 13 to 17 years^37,38,39,40^ with a higher prevalence in girls. The preinjury rates reported in this study were comparable for age-reported rates of these common comorbidities. The rate of migraine in our cohort may be higher than in the general population.^41,42,43^ In a post hoc analysis, our cohort demonstrated higher rates of migraine in adolescents and significant interaction between sex and age, with adolescent girls and women having the highest rate.

The comorbidities of emotional distress (defined here as depression or anxiety) and migraine were both associated with longer recoveries, which mirrors findings in other cohorts.^7,44^ We found that girls and women were more likely to report unchanged or worsening symptoms over the first week compared with boys and men, who were more likely to report improving symptoms over this time window.

While girls and women were at greater risk for prolonged recovery, the association of comorbid emotional distress or migraine to recovery were independent of sex. This suggests that comorbidities do not account entirely for the sex differences in symptoms and recovery seen at longer time windows after concussion and that some diagnoses, like migraine, anxiety, and depression, may have underlying biological characteristics that prolong symptom recovery in both sexes. Because these conditions are treatable, early identification may provide means to accelerate recovery. This may have important implications for initial assessment and potential interventions to prevent or treat PPCS.

### Limitations

This study has some limitations. While data was collected from 3 different institutions and health care settings, our cohort contained a high proportion of White, well-insured patients with highly educated parents. This suggests that our population may be less generalizable to the general population, and greater outreach is needed, as all 3 institutions treat patients regardless of insurance status. Nonetheless, this is a large prospective study of concussion and recovery in a subspecialty clinic population.

The 4CYC is a unique consortium of multidisciplinary centers, which differs from many earlier studies using ED, primary care, or sports concussion cohorts. This limits the acute injury severity details available; however, acute injury severity has been shown to be a weak estimator of prolonged recovery.^34^ Visits outside of the 4CYC specialty clinics were not captured, limiting generalization.

Collecting recovery data for concussion is a challenge. A large proportion of patients with mild TBI recover over time and may not return for follow up. Our study addressed this challenge by disseminating surveys to the patient’s parents every 3 months after the injury date to capture a recovery date without the need for a follow-up visit. We had good response rates for the follow-up survey. The only difference between the group who responded to follow-up and those who did not was that the group who responded to follow-up had fewer comorbidities. Most recovery times were determined by the clinician at a follow-up visit. While different factors might influence parent report of recovery, these data were collected prospectively with a uniform definition of complete recovery to minimize potential bias. Additionally, the comorbidity data in this study were reported by the parent and patient in medical interviews by a licensed clinician as part of normal clinical care but not necessarily independently diagnosed by the clinician.

## Conclusions

The 4CYC is a multicenter group organized to prospectively study the subspecialty clinic presentation and recovery of a pediatric patients with mild TBI. A substantial proportion of patients in this cohort study experienced prolonged recovery. Sex differences in recovery time were observed, with girls and women taking longer to recover than boys and men. Patients reporting comorbidities of emotional distress (ie, anxiety or depression) and migraine recovered more slowly, independent of sex. Understanding factors associated with prolonged recovery and PPCS in pediatric patients with mild TBI is essential to accurate prognostic estimates and to identify phenotypes for which specific therapeutic interventions can be applied more effectively.

## Notes

Supplement. **eTable 1.** Comparison of Patients Who Consented for Follow-Up vs Those Who Did Not **eTable 2.** Comparison of Patients with Complete Data vs Those Who Were Lost to Observational Follow-Up **eTable 3.** Event Characteristics by Sex **eTable 4.** Event Characteristics by Age **eTable 5.** Acute Injury Severity Surrogates **eTable 6.** Sex and History of Emotional Distress Interaction **eTable 7.** Sex and History of Migraine Interaction
