# Supplementary material for: Characteristics of Pediatric Mild Traumatic Brain Injury and Recovery in a Concussion Clinic Population
Source: JAMA Netw Open. 2020 Nov 16;3(11):e2021463. doi: 10.1001/jamanetworkopen.2020.21463 (PMC7670312; doi:10.1001/jamanetworkopen.2020.21463)
Supplement: Supplement. — eTable 1. Comparison of Patients Who Consented for Follow-Up vs Those Who Did Not eTable 2. Comparison of Patients with Complete Data vs Those Who Were Lost to Observational Follow-Up eTable 3. Event Characteristics by Sex eTable 4. Event Characteristics by Age eTable 5. Acute Injury Severity Surrogates eTable 6. Sex and History of Emotional Distress Interaction eTable 7. Sex and History of Migraine Interaction [file jamanetwopen-e2021463-s001.pdf]

Supplemental Online Content

Rosenbaum PE, Locandro C, Chrisman SPD, et al. Characteristics of pediatric mild traumatic brain injury and recovery in a concussion clinic population. *JAMA Netw Open.* 2020;3(11):e2021463. doi:10.1001/jamanetworkopen.2020.21463

eTable 1. Comparison of Patients Who Consented for Follow-Up vs Those Who Did Not
eTable 2. Comparison of Patients with Complete Data vs Those Who Were Lost to Observational Follow-Up
eTable 3. Event Characteristics by Sex
eTable 4. Event Characteristics by Age
eTable 5. Acute Injury Severity Surrogates
eTable 6. Sex and History of Emotional Distress Interaction
eTable 7. Sex and History of Migraine Interaction

This supplemental material has been provided by the authors to give readers additional information about their work.

© 2020 Rosenbaum PE et al. *JAMA Network Open.*

eTable 1. Comparison of Patients Who Consented for Follow-Up vs Those Who Did Not

	Eligibility and consent status		
	**Waiver of consent (N = 228)**	**Consented (N = 372)**	**P-value**
**Age at injury**			0.301[1]
5 - 12 years old	57 (25.0%)	108 (29.0%)	
13 - 18 years old	171 (75.0%)	264 (71.0%)	
**Sex**			0.064[1]
Male	116 (50.9%)	160 (43.0%)	
Female	112 (49.1%)	212 (57.0%)	
**Total number of comorbidities (excluding seizures)**			0.586[2]
0	51 (22.4%)	84 (22.6%)	
1	76 (33.3%)	122 (32.8%)	
2	25 (11.0%)	45 (12.1%)	
3 or more	23 (10.1%)	45 (12.1%)	
Unknown	53 (23.2%)	76 (20.4%)	

[1] Fisher's exact test.
[2] Kruskal-Wallis test.

© 2020 Rosenbaum PE et al. *JAMA Network Open.*

eTable 2. Comparison of Patients with Complete Data vs Those Who Were Lost to Observational Follow-Up

	Respond		
	**No (N = 73)**	**Yes (N = 271)**	**P-value**
**Age at injury**			0.240[1]
5 - 12 years old	16 (21.9%)	80 (29.5%)	
13 - 18 years old	57 (78.1%)	191 (70.5%)	
**Sex**			0.894[1]
Male	30 (41.1%)	116 (42.8%)	
Female	43 (58.9%)	155 (57.2%)	
**Total number of comorbidities (excluding seizures)**			0.008[2]
0	8 (11.0%)	71 (26.2%)	
1	21 (28.8%)	90 (33.2%)	
2	13 (17.8%)	30 (11.1%)	
3 or more	10 (13.7%)	31 (11.4%)	
Unknown	21 (28.8%)	49 (18.1%)	

[1] Fisher's exact test.
[2] Kruskal-Wallis test.

© 2020 Rosenbaum PE et al. *JAMA Network Open.*

eTable 3. Event Characteristics by Sex

	Sex			
	**Male (N = 276)**	**Female (N = 324)**	**Overall (N = 600)**	**P-value**
**Time to initial clinic visit (days)**				0.876[1]
N	276	324	600	
Median [Q1, Q3]	16.5 [8.5,29.0]	16.0 [8.0,29.0]	16.0 [8.0,29.0]	
**Definite reported mechanism of injury**				0.517[2]
Yes (There was a discrete event with force to the head or rapid head movement without impact)	271 (98.2%)	315 (97.2%)	586 (97.7%)	
No (There was no discrete event)	3 (1.1%)	6 (1.9%)	9 (1.5%)	
Unknown	2 (0.7%)	3 (0.9%)	5 (0.8%)	
**Gradual recovery of symptoms over the first week of the injury**				0.001[2]
Yes (There have been improving symptoms over the first week)	164 (59.4%)	142 (43.8%)	306 (51.0%)	
Yes (There have been stable symptoms over the first week)	88 (31.9%)	143 (44.1%)	231 (38.5%)	
No (There have been worsening symptoms over the first week)	21 (7.6%)	32 (9.9%)	53 (8.8%)	
Unknown	3 (1.1%)	7 (2.2%)	10 (1.7%)	
**Alternative explanation for the acute onset of symptoms**				0.766[2]
Yes (acute alternative conditions: migraine, cervical injury, heatstroke, syncope, panic attack, intercurrent illness, etc)	20 (7.2%)	30 (9.3%)	50 (8.3%)	
No (concussion is the only likely cause for the acute onset of symptoms)	241 (87.3%)	278 (85.8%)	519 (86.5%)	
Unknown	2 (0.7%)	4 (1.2%)	6 (1.0%)	
**Mechanism/cause of injury**				0.366[2]
Motor vehicle crash (MVC)	11 (4.0%)	17 (5.2%)	28 (4.7%)	
Assault	6 (2.2%)	6 (1.9%)	12 (2.0%)	
Struck by or against object/person (non-assault, non-sport)	20 (7.2%)	35 (10.8%)	55 (9.2%)	
Fall (non-sport)	26 (9.4%)	21 (6.5%)	47 (7.8%)	
Sport/Recreation	210 (76.1%)	242 (74.7%)	452 (75.3%)	
Unknown	3 (1.1%)	3 (0.9%)	6 (1.0%)	
**Retrograde Amnesia**	39 (14.1%)	40 (12.3%)	79 (13.2%)	0.390[2]
**Anterograde Amnesia**	61 (22.1%)	55 (17.0%)	116 (19.3%)	0.091[2]

© 2020 Rosenbaum PE et al. *JAMA Network Open.*

	Sex			
	**Male (N = 276)**	**Female (N = 324)**	**Overall (N = 600)**	**P-value**
**Loss of consciousness**	39 (14.1%)	30 (9.3%)	69 (11.5%)	0.052[2]

[1] Wilcoxon rank-sum test.
[2] Fisher's exact test.

© 2020 Rosenbaum PE et al. *JAMA Network Open.*

eTable 4. Event Characteristics by Age

	Age Category			
	5 - 12 years old (N = 165)	13 - 18 years old (N = 435)	Overall (N = 600)	P-value
**Time to initial clinic visit (days)**				0.736[1]
N	165	435	600	
Median [Q1, Q3]	17.0 [9.0,29.0]	16.0 [8.0,29.0]	16.0 [8.0,29.0]	
**Definite reported mechanism of injury**				1.000[2]
Yes (There was a discrete event with force to the head or rapid head movement without impact)	162 (98.2%)	424 (97.5%)	586 (97.7%)	
No (There was no discrete event)	2 (1.2%)	7 (1.6%)	9 (1.5%)	
Unknown	1 (0.6%)	4 (0.9%)	5 (0.8%)	
**Gradual recovery of symptoms over the first week of the injury**				0.467[2]
Yes (There have been improving symptoms over the first week)	90 (54.5%)	216 (49.7%)	306 (51.0%)	
Yes (There have been stable symptoms over the first week)	57 (34.5%)	174 (40.0%)	231 (38.5%)	
No (There have been worsening symptoms over the first week)	14 (8.5%)	39 (9.0%)	53 (8.8%)	
Unknown	4 (2.4%)	6 (1.4%)	10 (1.7%)	
**Alternative explanation for the acute onset of symptoms**				0.537[2]
Yes (acute alternative conditions: migraine, cervical injury, heatstroke, syncope, panic attack, intercurrent illness, etc)	14 (8.5%)	36 (8.3%)	50 (8.3%)	
No (concussion is the only likely cause for the acute onset of symptoms)	145 (87.9%)	374 (86.0%)	519 (86.5%)	
Unknown	2 (1.2%)	4 (0.9%)	6 (1.0%)	
**Mechanism/cause of injury**				<.001[2]
Motor vehicle crash (MVC)	8 (4.8%)	20 (4.6%)	28 (4.7%)	
Assault	4 (2.4%)	8 (1.8%)	12 (2.0%)	
Struck by or against object/person (non-assault, non-sport)	25 (15.2%)	30 (6.9%)	55 (9.2%)	
Fall (non-sport)	26 (15.8%)	21 (4.8%)	47 (7.8%)	
Sport/Recreation	100 (60.6%)	352 (80.9%)	452 (75.3%)	
Unknown	2 (1.2%)	4 (0.9%)	6 (1.0%)	
**Retrograde Amnesia**	17 (10.3%)	62 (14.3%)	79 (13.2%)	0.483[2]
**Anterograde Amnesia**	35 (21.2%)	81 (18.6%)	116 (19.3%)	0.187[2]

© 2020 Rosenbaum PE et al. *JAMA Network Open.*

	Age Category			
	5 - 12 years old (N = 165)	13 - 18 years old (N = 435)	Overall (N = 600)	P-value
**Loss of consciousness**	23 (13.9%)	46 (10.6%)	69 (11.5%)	0.142[2]

[1] Wilcoxon rank-sum test.
[2] Fisher's exact test.

© 2020 Rosenbaum PE et al. *JAMA Network Open.*

eTable 5. Acute Injury Severity Surrogates

	Overall (N = 600)
**Imaging done**	
CT and MRI	9 (1.5%)
CT	105 (17.5%)
MRI	14 (2.3%)
Unknown	35 (5.8%)
None	434 (72.3%)
Unknown	3 (0.5%)
**Findings of the CT**	(N=114)
Normal	92 (80.7%)
Abnormal	18 (15.8%)
Unknown	4 (3.5%)
**Findings of the MRI**	(N=23)
Normal	18 (78.3%)
Abnormal	5 (21.7%)
**Retrograde Amnesia**	
No	444 (74.0%)
Yes	79 (13.2%)
Unknown	77 (12.8%)
**Length of retrograde amnesia**	(N=79)
≤ 1 minute	8 (10.1%)
> 1 minute - 20 minutes	4 (5.1%)
> 20 minutes - 1 hour	9 (11.4%)
> 1 hour - 24 hours	11 (13.9%)
> 1 day - 7 days	8 (10.1%)
More than 7 days	12 (15.2%)
Unknown	27 (34.2%)
**Anterograde Amnesia**	
No	410 (68.3%)
Yes	116 (19.3%)
Unknown	74 (12.3%)
**Length of anterograde amnesia**	(N=116)

© 2020 Rosenbaum PE et al. *JAMA Network Open.*

	Overall<br>(N = 600)
≤ 1 minute	8 (6.9%)
> 1 minute - 20 minutes	18 (15.5%)
> 20 minutes - 1 hour	16 (13.8%)
> 1 hour - 24 hours	18 (15.5%)
> 1 day - 7 days	12 (10.3%)
More than 7 days	10 (8.6%)
Unknown	34 (29.3%)
**Loss of consciousness (LOC)**	
No	472 (78.7%)
Yes	69 (11.5%)
Unknown	59 (9.8%)
**Length of LOC**	(N=69)
Less than 1 minute	38 (55.1%)
Between 1-5 minutes	14 (20.3%)
Greater than 5 minutes	5 (7.2%)
Unknown	12 (17.4%)

CT: computerized tomography; MRI: magnetic resonance imaging

© 2020 Rosenbaum PE et al. *JAMA Network Open.*

eTable 6. Sex and History of Emotional Distress Interaction

Obs	Parameter	ClassVal0	ClassVal1	DF	Estimate	StdErr	ChiSq	ProbChiSq	HazardRatio	Label
1	Gender	Female		1	-0.12998	0.22861	0.3233	0.5697	.	Gender Female
2	History of Emotional Distress	No		1	0.34848	0.20439	2.9070	0.0882	.	Mental Distress No
3	Gender*History of Emotional Distress	Female	No	1	-0.05769	0.25940	0.0495	0.8240	.	Gender Female * Mental Distress No

© 2020 Rosenbaum PE et al. *JAMA Network Open.*

eTable 7. Sex and History of Migraine Interaction

Obs	Parameter	ClassVal0	ClassVal1	DF	Estimate	StdErr	ChiSq	ProbChiSq	HazardRatio	Label
**1**	Gender	Female		1	-0.42414	0.30227	1.9690	0.1606	.	Gender Female
**2**	HistoryMigraines	No		1	0.33680	0.25738	1.7123	0.1907	.	Migraines No
**3**	Gender*HistoryMigrai	Female	No	1	0.21084	0.32383	0.4239	0.5150	.	Gender Female * Migraines No

© 2020 Rosenbaum PE et al. *JAMA Network Open.*
